# Personalised Interventions—A Precision Approach for the Next Generation of Dietary Intervention Studies

**DOI:** 10.3390/nu9080847

**Published:** 2017-08-09

**Authors:** Baukje de Roos, Lorraine Brennan

**Affiliations:** 1 The Rowett Institute, University of Aberdeen, Foresterhill, Aberdeen AB25 2ZD, UK; 2 Institute of Food and Health and Conway Institute, UCD School of Agriculture and Food Science, UCD, Belfield, Dublin 4, Ireland; lorraine.brennan@ucd.ie

**Keywords:** precision nutrition, personalised nutrition, dietary efficacy, bioavailability, dietary advice, metabolomics, phenotyping, nutrigenetics

## Abstract

Diet is a key modifiable risk factor for non-communicable diseases. However, we currently are not benefitting from the full potential of its protective effects. This is due to a number of reasons, including high individual variability in response to certain diets. It is now well acknowledged that in order to gain the full benefit of dietary regimes it is essential to take into account individual responses. With this in mind, the present review examines the concept of precision nutrition and the performance of n-of-1 studies, and discusses the development of certain approaches that will be critical for development of the concepts.

## 1. Diet, Dietary Efficacy, and Dietary Advice

Dietary risk factors are currently the most important risk factor for the Global Burden of Disease—globally, in 2013, dietary risks accounted for 11.3 million deaths and 241.4 million disability-adjusted life-years. Dietary risks include a diet low in vegetables, low in seafood, low in whole grains, low in nuts/seeds, low in milk, and high in red meat [1]. Furthermore, nutritional practices have been shown to reduce the risk of cardiovascular disease by 60% [2], and it is estimated that 27–39% of the main cancers can be prevented by improving diet, physical activity, and body composition [3]. The efficacy by which dietary interventions influence health is currently mainly determined by taking population-based approaches that can favourably shift disease risk factors in the entire population [4]. However, these approaches may be ineffective as two major issues are overlooked.

First of all, although intuitively appealing to try and encourage healthy food choice through information provision and education, knowledge alone is typically insufficient to change behaviour. While the vast majority of people know what they ‘should’ and ‘should not’ eat, turning intentions into action is notoriously difficult. Less than a quarter of all people who embark on a healthy eating plan still stick to it 12 months later [5]. Laboratory studies demonstrate that self-control resources are finite and people have a limited amount of the mental energy required to resist temptation [6]. In most European countries, eating styles do not match with basic recommendations. Despite some improvements, diets still contain too much saturated fat, sugar, and salt, and insufficient vegetables, fruits, and fish [7]. This is specifically problematic for those already suffering from diseases where diet plays an important role in its aetiology, like metabolic diseases. Highly-perceived susceptibility to disease is, however, known to influence motivation for individual behaviour change [8], and therefore, diets tailored to individual circumstances may prove more effective.

Secondly, it is now becoming clear that there is considerable inter-individual variation in response to dietary interventions, and some interventions may benefit certain individuals or population subgroups more than others, depending on their genotype, phenotype, and environment [9,10]. Many of the large, randomised controlled trials have effectively demonstrated that only 40% of a cohort responds to the dietary interventions [11], and lifestyle measures that reduce disease risk in an entire population may offer little benefit to a specific individual. This means that for the same dietary intake, exposure to bioactive metabolites can markedly differ between individuals. For example, the inter-individual variation in response to eating beneficial plant food bioactives may result from a range of determinants including age, sex, habitual dietary habits, genetics, epigenetics, and gut microbiota. This will affect the absorption, distribution, metabolism, and excretion of compounds and metabolites, and thus affect biovailability and biological responsiveness [12]. For example, ‘responders’ and ‘non-responders’ to carotenoids, classified as subjects with high or low carotenoid status upon consumption, respectively, may be explained by variants of genes encoding proteins involved in the absorption and metabolism of carotenoids, such as SR-BI (scavenger receptor class B, member 1) and CD36 (CD36 molecule, thrombospondin receptor), which are involved in the uptake of carotenoids by intestinal cells, as well as BCO1 (beta-carotene oxygenase 1), which catalyses the oxidative cleavage of carotenoids into vitamin A [13]. Gut microbiota are known to play an important role in efficacy of the soy isoflavone diadzin/daidzein. This plant bioactive is converted into equol by gut microbiota but only in 25–35% of the Western population and up to 50–70% of the vegetarian and Asian populations [14,15]. In one of our recent controlled clinical trials we showed that consumption of flavan-3-ol-enriched dark chocolate decreased platelet aggregation in males but not female subjects. This effect could not be correlated with plasma or urine concentrations of flavan-3-ols metabolites, suggesting that inter-individual variation in biological responses is not always due to variation in the bioavailability of plant food bioactives but can depend on other factors also, such as sex [16].

## 2. Precision Medicine and Precision Nutrition

**Figure 1:**
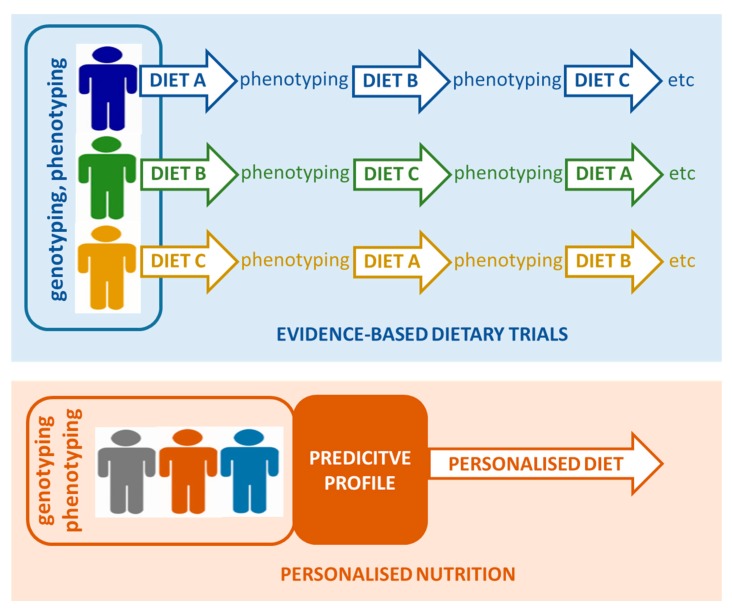
Concept of n-of-1 trials leading to personalised interventions.

This phenomenon of inter-individual variability is not unique for nutrition sciences–medical sciences have pioneered the introduction of Precision Medicine in an attempt to overcome and indeed benefit from individualised/variable response to therapies. Recently, there has been significant acceptance of the fact that the medical field needs to take individual variability into account, and indeed, there has been a recent call for the development of n-of-1 clinical trials that focus on the individual, and not average responses to medical therapy [17,18,19]. Furthermore, regulatory agencies such as the US Food and Drug Administration (FDA) are also recognising the importance of individual responses. These so called n-of-1 trials focus on a single person with data collected over a time-course as the individual undergoes different treatments (see Figure 1). Aggregated results of many n-of-1 trials offer data on how to better treat sub-populations that share genetic and phenotypic factors, amongst others, or the population at large [20,21]. Such trials can also employ omics-based technologies and bioinformatics, so as to analyse and link the dynamics of thousands of molecules and parameters in a single individual, and understand the differences of these dynamics between different individuals and subgroups, providing a deeper understanding of dynamic changes in molecular components and biological pathways across different health conditions and individuals. Indeed, monitoring of the omics profiles of an individual has already been shown to be a useful tool in the dynamic assessment of the physiology and health of an individual [22,23,24]. This approach has been coined as iPOP (integrative personal omic profiling) and is set to identify new pathways and genes that are related to disease development.

In the field of personalised nutrition there have been a number of studies that have demonstrated the potential of tailoring dietary advice to the individual level. Combining continuous monitoring of glucose and omics techniques in an 800-person cohort, Zeevi and colleagues developed a strategy for the prediction of glycemic responses to meals. To achieve this they monitored the responses to almost 47,000 meals which demonstrated a high variability in responses to identical meals. A machine-learning algorithm was applied to integrate dietary habits, anthropometric outcomes, physiological parameters in blood, levels of physical activity, and gut microbiota in order to develop a prediction model for the glycaemic response to real life meals. This prediction model was subsequently tested in an independent cohort of 100 persons. Finally, a randomised, controlled dietary intervention in 26 participants showed that the algorithm rightly predicted significantly lower postprandial responses for specific meals [9]. In a pan-European Study the Food4Me personalised intervention demonstrated that tailoring dietary advice to an individual level resulted in an improvement in the dietary quality of the participants, providing evidence for the efficacy of the approach [25]. A recent review further highlighted the emerging field of n-of-1 studies for precision nutrition [26].

## 3. Adaptation of Precision Nutrition Approaches in Future Studies

The application of precision nutrition approaches has the potential to significantly affect the way we perform dietary intervention studies in the future. In addition, improved knowledge of individual factors that determine the bioavailability and efficacy of food components could improve the way we formulate dietary advice. Indeed, in the future this may lead to personalised and tailored dietary advice for individuals or population subgroups for foods or food groups. However, in order to make that transition from population-based guidelines to individual dietary advice, we will require the execution of many more carefully designed precision nutrition trials. Other factors that will determine the success of the implementation of precision nutrition approaches in future studies include the expansion of the field of metabolomics to allow deep phenotyping of baseline status and responses to dietary inventions, and an in-depth understanding of the role of nutrigenetics in the inter-individual responses to food components and products. More recently, evidence is beginning to emerge of the role of the gut microbiota in individualised responses to dietary interventions, thus highlighting the possibility of the use of gut microbial analysis as a method for tailoring dietary advice [27,28].

### 3.1. Phenotyping of Individuals to Enable Precision Nutrition

A key aspect that will be essential for the development of precision nutrition will be the use of nutrigenomic approaches to allow phenotyping at the individual level. In recent years the application of metabolomics has emerged as a powerful tool in the identification of individual responses to drug therapies [29,30,31], and the concept of metabotypes has emerged where different metabotypes display differential responses. A similar concept holds true for nutrition with the identification of metabotypes that characterise differential responses to nutrition interventions [32,33]. The importance of such approaches lies in the identification of a biomarker signature that could be used to deliver tailored dietary advice.

An important area where metabolomics is set to play an important role is the development of biomarkers that are related to the efficacy of a certain dietary regime. 

Currently, prognostic and diagnostic biomarkers are used to assess the efficacy of a diet or bioactive food compound in the short or longer term. Both types of biomarkers are indicators for a disorder that has already developed, which is very relevant for a chronic disease like cardiovascular disease or type 2 diabetes, which have the potential to start developing in the first decade of life [34]. Several biomarkers and criteria have been proposed to validate the influence of food components on specific physiological functions, such as type 2 diabetes [35] and cardiovascular disease [36]. The application of prognostic and diagnostic biomarkers in human intervention studies, however, is often not straight forward for at least three reasons. Firstly, our current collection of classic or new biomarkers is clearly not adequate for explaining how diet or food compounds can decrease cardiovascular disease risk, especially in relation to pathways involved in inflammation and oxidative stress. Secondly, although the classical and new biomarkers might be perfectly able to indicate the risk of cardiovascular disease in patients, or identify modification of risk through pharmacological intervention, such markers may not always be appropriate for subtle dietary interventions in subjects that are relatively healthy. Thirdly, the link between diet and chronic diseases is complex and difficult to unravel. Our diet is made up of many different food compounds and nutrients, and most of these may uniquely affect risk of developing vascular disease. Only when we address all these issues we can seriously progress with the measurement of the efficacy of dietary nutrients to benefit health [37].

The development of biomarkers of health is even more challenging. The World Health Organization (WHO) defined health in 1948 as “a state of complete physical, mental, and social well-being and not merely the absence of disease or infirmity” [38], and based on this, it has been proposed that the formulation of health should be the “ability to adapt and to self-manage” [39]. Therefore, the instruments to measure health should not only be those markers we use to assess the risk of developing chronic diseases, such as blood pressure, plasma lipids, and blood glucose. Instead, markers to measure health should relate to resilience and the ability to cope with stresses to the system that prevent disease from happening in the first place [40]. With this in mind, the concept of the challenge test has emerged where an individual′s response to a meal challenge is monitored and biomarkers indicative of a healthier response are beginning to emerge [41,42]. The use of such biomarkers to guide the delivery of personalised nutrition advice has significant opportunity but needs further proof of concept studies. 

A further aspect that is equally important in the delivery of precision nutrition is the development of tools to assess dietary intake and nutrition status. Diet is a complex, multi-dimensional exposure, and its assessment requires a multipronged approach which may include high-throughput nutritional metabolomics complementary to traditional assessment tools such as validated dietary questionnaires and established nutrient biomarkers. To achieve the goal of precision nutrition, more efforts are needed to develop, validate, and refine assessment methods that can capture the multidimensional nature of diet [43]. Exciting new developments in the area of point-of-care diagnostics promise improved access to nutritional status assessment, which would be a first step towards tailored interventions. However, a systematic evaluation of their accuracy, reproducibility, and sensitivity is mostly lacking [44]. With the application of metabolomics, new biomarkers of dietary intake are emerging [45]. Importantly, we have recently demonstrated that such biomarkers are capable of determining dietary intake at the g/day level [46]. Using a well-controlled feeding study we developed calibration curves between the biomarker urinary level and the actual amount of food consumed (citrus fruit). Using these calibration curves we were able to determine the citrus fruit intake in a population study using the biomarker proline betaine. The importance of this lies in the potential use of combinations of biomarkers to determine the intake of important foods in the diet. Furthermore, combining dietary biomarkers with the classical approaches has the potential to improve our ability to accurately assess dietary intake. Indeed, in recent years, research on the development and validation of methods of online recording of food intake has expanded significantly [47]. However, the accurate monitoring of dietary intake, especially those based on web-based tools, remains a significant challenge, especially when applying such tools across countries due to the variety in dietary patterns and available food databases.

In addition to intake, exposure to food components should be taken into account when trying to link dietary nutrients and patterns to health or disease development. Factors such as gender, age and host genetic, and epigenetic variations in metabolising enzymes cause major differences in the actual bioavailability of nutrients between people, as discussed above. More advanced metabolomic assessment of individuals after the consumption of specific diets, in relation to information obtained from dietary assessment tools, is expected to provide novel biomarkers for a range of food exposures. Dried blood spots (DBS) are a promising tool to assess dietary intake and the nutritional status of certain dietary components in population-based epidemiology studies, because DBS can be collected by non-phlebotomists in non-clinical settings, and are more easily transported and stored. However, DBS methods need to be carefully developed and validated against venous methods [48,49]. Overall, with the development of the latest nutrigenomic technologies, their potential use in the delivery of precision nutrition grows. While more work is needed in the development of aspects such as the biomarkers of health, it is also imperative that we commence testing the n-of-1 approach and demonstrate its utility.

### 3.2. Nutrigenetics

Lifestyle interventions, including dietary interventions, which alter the health or disease status against a background of genetic variability, are identified mainly via population-based approaches in genome-wide association studies (GWAS). For common complex diseases, however, the GWAS-driven advances in the annotation of our genetic architecture over the past decade have not led to a concomitant shift in refined treatments. Similarly, attempts to disentangle treatment responders from non-responders via genetic predictors have not met their anticipated success [50].

Determining whether corresponding changes in diet in turn favourably shift disease risks requires appropriately designed prospective studies. In recent last decades, hundreds of nutrigenetics studies have attempted to establish the role of single SNPs in explaining inter-individual variability in response to diets and nutrients. Whilst important to do, we have learned little in terms of the overall impact on health. Most nutrigenetics studies are based on associations only–in these cases, a higher response to a specific dietary factor or nutrient was observed for a particular genotype at a candidate gene locus. However, genetic association studies often have limited statistical power and also frequently lack reproducibility. Indeed, most studies are unique and findings have not been confirmed by others. Thus far only two studies were designed such that subjects were recruited prospectively based on genotype. One multi-centre double-blind placebo-controlled human intervention study in 312 adults assessed the effects of fish oil supplements on APOE-genotype dependent changes in plasma triglycerides levels (FINGEN study). The investigators observed that whilst plasma triacylglycerol concentrations were lowered in the group as a whole, the greatest triacylglycerol-lowering responses were evident in men carrying the apolipoprotein E4 (*APOE4*) variant [51]. A second study investigated the effect of riboflavin, the cofactor for methylenetetrahydrofolate reductase (MTHFR), on blood pressure in patients who were homozygous for the 677C→T polymorphism (TT genotype) in the gene encoding for MTHFR. In this placebo-controlled crossover study, patients with the TT genotype had higher systolic blood pressure at baseline, and the riboflavin supplementation produced an overall significant and physiologically relevant decrease in systolic and diastolic blood pressure [52]. Apart from these two studies, no other available studies give conclusive evidence for the role of distinct single SNPs in the individual response to dietary changes or nutrient status. 

It could be argued that single genotypes have generally poor predictive value for health outcomes. However, in almost all complex diseases and in particular in the non-communicable diseases, a few tens or even hundreds of genes are involved in a phenotypic trait. Moreover, downstream processes such as epigenetics, miRNA interference, and other forms of posttranslational alterations of protein functions will affect the proteome and metabolome, and are thus contributing substantially to the phenotype. The importance of these effectors is just starting to emerge from large scale cohort studies.

This all shows that the current state of nutrigenetics is currently of limited value for an individual or the public in guiding healthy nutrition. Therefore, it would be premature to apply our knowledge in the field of nutrigenetics to personalised diets for specific genotypes [53]. This observation was confirmed recently in a large proof-of-principle study in a pan-European cohort (www.Food4Me.org) to establish the effectiveness of an internet-based personalised nutrition approach in improving dietary behaviours. Participants randomised to the personalised nutrition arms of the intervention study had an overall increase in the healthy eating index, lower salt intake, and lower intake of red meat based on general healthy dietary advice and/or knowledge of individual nutrient status. Importantly, including genotypic information in the development of the personalised nutrition advice did not produce any additional benefit [25]. 

## 4. Conclusions

An increasing number of studies indicate the need to apply precision nutrition approaches to personalise disease risk and, in parallel, future dietary recommendations. Further developments in this area will depend on big data analysis and health informatics that can capture molecular and medical data. Ultimately, the success of precision nutrition approaches will depend on the robust application of appropriate study designs as the predictive role of biomarkers cannot be definitively ascertained without randomly assigning subjects to some form of control treatment [54].
